# Goal Side Selection of Penalty Shots in Soccer: A Laboratory Study and Analyses of Men’s World Cup Shoot-Outs

**DOI:** 10.1177/00315125211025412

**Published:** 2021-06-23

**Authors:** Mauro R. Pereira, Geoffrey R. Patching

**Affiliations:** 1 Department of Psychology, 5193 Lund University, Lund University, Lund, Sweden

**Keywords:** soccer, penalty kick, line bisection, landmark task, Bayesian modelling

## Abstract

Penalty kicks in soccer provide a unique scenario in which to examine human choice behavior under competitive conditions. Here, we report two studies examining the tendency for soccer kickers to select the goal side with the largest area to the left or right of the goalkeeper’s veridical midline, when the goalkeeper stands marginally off-center. In Study I participants viewed realistic images of a soccer goal and goalkeeper with instructions to choose the left or right side of the goalmouth to best score a goal. We systematically displaced the goalkeeper’s position along the goal line; and, to simulate changes in the kicker’s viewing position, we systematically displaced the lateral position of the goalmouth in each image. While, overall, participants tended to choose the left over the right goal side, this preference was modulated by the goalkeeper’s position relative to the center of the goal and jointly on the lateral position of the goalmouth relative to the participants’ body midline. In Study II we analyzed 100 penalty shots from men’s world cup shoot-outs between the years 1982 to 2018. Again, we found a small tendency for kickers to aim the ball to the left goal side, but with barely any modulating effect of changes in the goalkeeper’s position and no effect of changes in the kicker’s position. In contrast to earlier claims that a goalkeeper may benefit by standing marginally to the left or right of the center of the goal to influence the direction of the kicker’s shot, our findings suggest that this is probably not a good strategy in elite football competitions.

## Introduction

The work reported in this article concerns goal side selection of penalty shots in a laboratory soccer experiment and in men’s FIFA World Cup matches. Inspiration for this work derives from studies of the so-called ‘off-center’ effect in penalty kick scenarios – the tendency for penalty kickers to place the ball to the goal side with the greater area to the side of the goalkeeper, when the goalkeeper stands marginally off-center (Masters et al., 2007; Memmert et al., 2020; Noël et al., 2016; Noël, van der Kamp, & Memmert, 2015; Noël, van der Kamp, Weigelt, et al., 2015; Weigelt & Memmert, 2012; Weigelt et al., 2012). Here, an aim was to extend understanding of penalty kickers’ goal side selection in soccer by further examination of the influence of the goalkeeper’s position and by simultaneous examination of the initial starting, and hence viewing position of the kicker. To date, there has been minimal research examining the influence of the goalkeeper’s position and, simultaneously, the initial viewing position of the kicker, on the kicker’s goal side selection of penalty kicks. Yet, most kickers approach the ball at an angle that may in turn at least partially determine goal side selection. As a result, goal side selection of penalty kicks in soccer as potentially influenced by the goalkeeper’s position may also depend on the initial starting position of the kicker.

Penalty kicks are used in soccer for two reasons: (a) to determine the winner when the score is tied after regulation playing time, or (b) as a punishment against a team that falls foul of certain rules during the game. Successful penalty kicks often decide game wins (Bar-Eli et al., 2007), and so provide a unique setting by which to examine human choice behavior under competitive conditions (Avugos et al., 2020; Chiappori et al., 2002). In a penalty kick, the kicker shoots a stationary ball from a penalty mark, aligned central to the goalmouth and located 36 ft (10.97 meters) away from the goal line. Until the ball is kicked, the goalkeeper must stay on the goal line between the left and right goal posts, and no other players are allowed to intervene (IFAB,2019/20).

From an analysis of penalty kicks taken in elite soccer competitions Masters et al. (2007) found that for 96% of the penalty kicks the goalkeeper was positioned slightly off-center, and for 59% of the shots the kicker aimed the ball to the goal side with the greatest area. These observations led Masters et al., to suggest that goalkeepers may obtain a small advantage in penalty kick situations by standing marginally to the left or right of the center of the goalmouth, to influence the direction of penalty kicks to the goal side with the greatest area and dive strategically to the goal side with the greater area.

Subsequently, Masters et al. (2007) presented three experimental studies examining the possibility that minor displacements of the goalkeeper from central can influence the goal side selection of kickers’ penalty shots in soccer. In the first experiment, participants viewed a rectangular outline on a computer screen, representing the goalmouth, with a small filled block on the ‘goal-line’ representing the goalkeeper (the rectangle was scaled to 3% of normal goal size). Given small displacements of the filled block from central, participants judged the side of goal with the greatest area. In the second experiment, the block was replaced with an image of Oliver Khan (renowned former German goalkeeper), and the goal and goalkeeper were projected onto a screen scaled to 44% of normal size. In this case, participants kicked a ball from a penalty spot to the side with the greater area. In both experiments, participants were able to discriminate goal side differences in areas as small as 0.5%, which remained constant regardless of the scaling of the images. In the third experiment, instructions were to take a penalty kick only when the goalkeeper (i.e., Kahn) was standing in the center of the goal. Nonetheless, participants kicked the ball regardless of small displacements of the goalkeeper, and they kicked the ball to the goal side with the largest area at above chance levels for differences in areas between ±1.6% and ±3%. This off-center effect has subsequently been found for penalty kicks taken against photo realistic images of a soccer goal and goalkeeper, and in trials with regular soccer players on adult sized soccer pitches (Memmert et al., 2020; Noël et al., 2016; Noël, van der Kamp, & Memmert, 2015; Noël, van der Kamp, Weigelt, et al., 2015; Weigelt & Memmert, 2012; Weigelt et al., 2012). Consequently, the off-center effect appears to be a reasonably stable phenomenon that occurs both on and off the soccer pitch.

In soccer related tasks, in which participants have selected the goal side with the greatest area, the proportion of kicks to the goal side with the greatest area has been found to increase monotonically as the area to the side of the goalkeeper is increased (Masters et al., 2007; Memmert et al., 2020; Weigelt & Memmert, 2012; Weigelt et al., 2012). Penalty kickers (soccer players and soccer novices alike) have also been found to misjudge the central position of a goalkeeper (Masters et al., 2007; Memmert et al., 2020; Noël et al., 2016; Noël, van der Kamp, & Memmert, 2015; Noël, van der Kamp, Weigelt, et al., 2015; Weigelt & Memmert, 2012; Weigelt et al., 2012). When asked to position a goalkeeper in the center of the goalmouth, from a viewing distance behind the penalty spot, participants were found to place the goalkeeper just to the right of center on most (62%) trials, and in the true veridical center of the goal on very few trials (< 3%) or not all (Noël, van der Kamp, & Memmert, 2015; Noël, van der Kamp, Weigelt, et al., 2015). Moreover, Noël, van der Kamp, Weigelt, et al. (2015) found a small tendency for kickers to place the ball to the left of the goal midline, but other studies (Memmert et al., 2020; Weigelt & Memmert, 2012; Weigelt et al., 2012) have shown a small tendency for participants to make more kicks to the right, as compared to left, goal side area.

Goal side selection of penalty kicks in soccer is widely considered analogous to the neuropsychological task of horizontal line bisection, especially the Landmark Task of selecting which, left or right, segment of a pre-bisected line is longer (Masters et al., 2007; Noël, van der Kamp, Weigelt, et al., 2015; Weigelt & Memmert, 2012; Weigelt et al., 2012). Experimentally displacing a bisection mark along a horizontal line, in small units from left to right of central, monotonically increases the probability of a left-side longer judgement, describing a classic sigmoidal function (Gökaydin et al., 2017; Märker et al., 2019; McCourt & Olafson, 1997; Toraldo et al., 2004). From this sigmoidal function, it is possible to estimate the location of the bisection mark that predictively gives rise to an equal (50%) proportion of left and right longer judgments (Märker et al., 2019; McCourt & Olafson, 1997; Toraldo et al., 2004); termed, point of subjective equality (PSE). With the Landmark Task, neurologically healthy participants tend to make marginally more left, as compared to right, line longer judgements (Jewell & McCourt, 2000; McCourt & Olafson, 1997; Milner et al., 1992; Thomas et al., 2015). By scaling participant’s binary choices the PSE was found to be located just to the left of the exact center of the line (Märker et al., 2019; McCourt & Olafson, 1997; Toraldo et al., 2004), indicative of a tendency for participants to over-estimate the length of the left- as compared to the right-line segment (Toraldo et al., 2004). Likewise, when asked to bisect a horizontal line into two equal parts neurologically healthy participants tend to mis-bisect the line placing their midline mark marginally to the left of true center (Milner et al., 1992). Yet, systematic asymmetries in line bisection are known to vary in magnitude and direction with changes in line length (McCourt & Jewell, 1999; Nicholls et al., 2016), and also with changes in viewing distance (Longo et al., 2015; McCourt & Garlinghouse, 2000; Nicholls et al., 2016; Rinaldi et al., 2018).

Systematic asymmetries in line bisection are also known to vary with experimental manipulation of the egocentric spatial location of lines (Reuter-Lorenz et al., 1990; Rinaldi et al., 2018; Zago et al., 2017). In general, the further leftward a horizontal line is placed relative to the participant’s body midline, the more leftward the bisection error, but this overestimation of the length of left line segments can cross over to become rightward as lines are presented further rightward in space (Bultitude & Davies, 2006; McCourt & Jewell, 1999; Mennemeier et al., 2001; Rinaldi et al., 2018). Consequently, kickers’ goal side selection of penalty shots in soccer may not only be related to the (off-center) position of the goalkeeper but also related to the starting, and hence viewing, position of the kicker. To date, the joint influence of both the goalkeeper’s position and the kicker’s position on the kicker’s goal side selection has been largely ignored in studies of the off-center effect in soccer.

The present study followed naturally on from the work of Masters et al. (2007) and others (Memmert et al., 2020; Noël et al., 2016; Noël, van der Kamp, & Memmert, 2015; Noël, van der Kamp, Weigelt, et al., 2015; Weigelt & Memmert, 2012; Weigelt et al., 2012). We set out to examine the joint effect of manipulating both the goalkeeper’s position and the kicker’s viewing position on the kicker’s goal side selection of penalty shots in a soccer related experimental task (Study I). Additionally, we analyzed what actually took place with regard to kickers’ choices in actual world cup soccer shootouts (Study II).

## Method: Study I

### Description and Hypotheses

In line with Weigelt and Memmert (2012) and Weigelt et al. (2012), we presented participants with photo realistic images of a goal and goalkeeper, here scaled to 2% of real size. We presented the goalkeeper at seven different locations along the goal line from -5% left to +5% right in small units of about 1.67%. To mimic changes in the kicker’s viewing position, we aligned participants centrally to the computer monitor and presented the goalmouth at seven different displacements, relative to the center of the computer monitor, from -5% left to +5% right in units of 1.67%. In line with real soccer matches, we instructed participants to choose the (left or right) goal side to best score a goal. This contrasts with studies of the off-center effect that have instructed participants to select the goal side with the greatest area (Masters et al., 2007; Memmert et al., 2020; Weigelt & Memmert, 2012; Weigelt et al., 2012), and with studies using the Landmark task in which participants are instructed to select the longer (or shorter) line segment (see Jewell & McCourt, 2000, for a review). Nonetheless, we predicted that the proportion of left goal side selections would rise monotonically as the position of the goalkeeper was moved from left to right along the goal-line, and that selections would systematically depend on the joint position of both the goalkeeper and kicker. Regressing participants’ binary goal side selections on the goalkeeper’s position and kicker’s position, relative to the veridical center of the goalmouth, were planned to reveal the precise extent to which both the goalkeeper’s position and kicker’s viewing position influenced participants’ goal side selection in this soccer related experimental task.

### Participants

For Study I, we recruited 40 participants from Lund University’s student population (9 women, 31 men; age range: 20–50 years, *M_age_* = 26.6, *SD* = 5.9 years). All participants claimed to be right-handed and reported normal or corrected to normal vision. All but three participants claimed to be right-footed. None of the participants played soccer on a regular basis.

All participants were informed of the experimental procedure, their right to withdraw from the study at any time without consequence, and all participants provided signed consent before taking part in the study. The study did not involve any deception or involve any invasive or potentially dangerous methods. According to the Swedish Ethical Review Authority and the guidelines of Lund University, where the study was conducted, formal ethical approval was not required.

### Apparatus

We used a microcomputer (Fujitso Lifebook Series 5) running MATLAB (The MathWorks, Inc.) to run the experiment, controlling the stimulus presentation and timing with the Psychophysics Toolbox extensions (Brainard, 1997; Pelli, 1997). The pixel resolution of the video monitor was 1366×768, with a refresh rate of 60 Hz. Participants responded using the two vertical arrow keys, marked with red and green stickers, positioned at the bottom right of the microcomputer’s standard QUERTY keyboard. For goal side selection, participants used the index finger of their right hand to press the down arrow key, and the middle finger of their right hand to press the up arrow key.

### Stimuli

**Figure 1.**
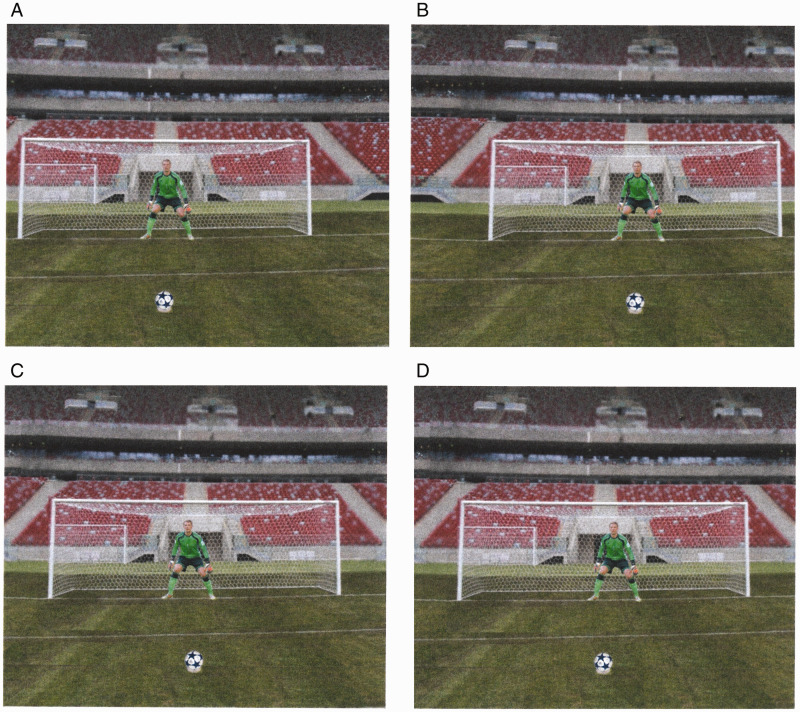
Example of Four Penalty Shootout Scenarios Used in the Experiment. *Note.* In all conditions, a picture of German goalkeeper (Manuel Neuer) was positioned on the goal line and a football was placed on the penalty spot. A: The goalkeeper is positioned centrally in the goal, and the goal mouth is offset −3.4 mm to the left of center, so from the egocentric viewpoint of the kicker (i.e., participant whose viewpoint was aligned with the center of the computer monitor), they are positioned 3.4 mm to the right of the ball. B: The goalkeeper is positioned centrally in the goal, and the goalmouth is offset 3.4 mm to the right of center, so the egocentric viewpoint of the kicker is −3.4 mm to the left of the ball. C: The goalkeeper is positioned −3.4 mm to the left of the center of the goal, and the egocentric viewpoint of the kicker is central in relation to the goalmouth. D: The goalkeeper is positioned 3.4 mm to the right of the center of the goal, and the egocentric viewpoint of the kicker is central in relation to the goalmouth.

The stimuli consisted of 16 images, each representing a unique condition characterized by different combinations of goalkeeper and goalmouth displacements. The goalkeeper was presented at seven different locations relative to the center of the goal, from −3.40 mm (left) to 3.40 mm (right) in six steps of 1.13 mm (i.e., −5% left to +5% right in small units of about 1.67%, ignoring rounding errors of no concern). In addition, the goalmouth was presented at seven different positions relative to the center of the computer monitor, from −3.40 mm (left) to 3.40 mm (right) in six steps of 1.13 mm. Each image was 185×156 mm in size. The goalmouth dimensions depicted in the images was 140×49 mm (0.0069 m^2^), which is 0.04% of the total area of original sized goals used in association football [7.32 m×2.44 m (17.86 m^2^)]. The goalkeeper’s height was 40 mm [approximately 2% of Manuel Neuer’s real height (1.93 m)], and the distance between the goal line and the penalty spot (where the ball was shown) was scaled to 0.3% (0.03 m) of real playing distance (11 m). Figure 1 shows four representative images.

Following guidelines from Masters et al. (2007), the goalkeeper’s position before the ball was kicked was the difference between the left and right goalmouth areas either side of the goalkeeper’s veridical midline, expressed as a percentage of the total goal mouth area, Δarea/area × 100 = (Left*_area_* – Right*_area_*)/Total*_area_* × 100. Likewise, the kicker’s position is expressed as the percentage difference in the left minus right goalmouth areas either side of the kicker’s body midline, before taking their run-up to kick the ball. In this respect, a −1% displacement of the player is equivalent to the player standing 3.4 cm to the left of the veridical center of the goalmouth on a regulation adult sized soccer pitch. Throughout, interpretation of the signed ± displacement of the goalkeeper’s position and kicker’s position from central is always from the kicker’s perspective, which is necessarily in opposition (inverse) to the goalkeeper’s perspective (what is right from the kicker’s perspective is left from goalkeeper’s perspective and vice versa).

### Design

**Figure 2.**
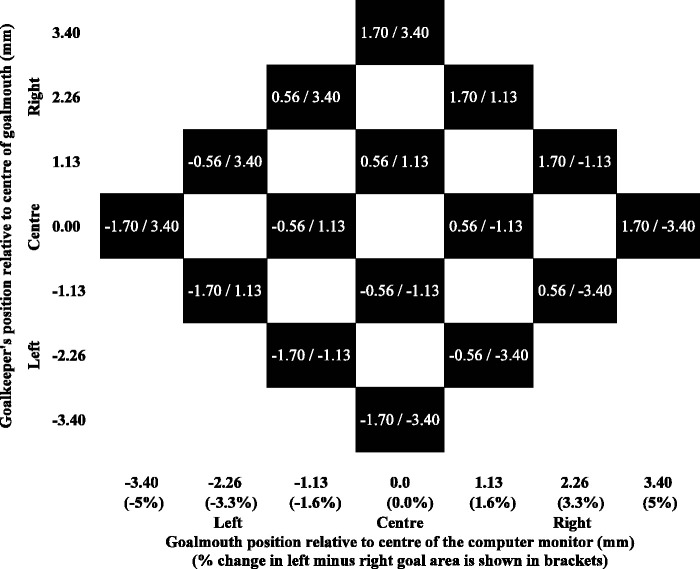
Semi-Factorial Combination of Stimuli Used in the Experiment. *Note.* The black squares show the pairings of the goalkeeper’s position (relative to the center of the goal) and goal mouth position relative to the center of the computer monitor. The lower left to upper right diagonal shows the mean position of the goalkeeper relative the center of the goal and goal mouth displacement relative to the center of the computer monitor. The opposing upper left to lower right diagonal shows the difference in the positions of the goalkeeper relative to the center of the goalmouth and goalmouth relative to the center of the computer monitor. There are some minor rounding errors of no concern.

Figure 2 illustrates the factorial combination of the goalkeeper’s positions and goalmouth displacements used in the experiment. Following Hellström (1978; Patching et al., 2012) the seven goalkeeper positions and seven goalmouth displacements were combined factorially about their mean position, and difference of position, to create 16 different stimuli in a diamond-shaped arrangement.

The experiment consisted of two sections (practice and experimental), with no break between the two sections. The first 32 trials, in which each stimulus was presented twice in pseudorandom order, were deemed practice trials. Following the practice trials, participants completed 256 experimental trials in which all the stimuli were presented 16 times in pseudo randomized cycles of 64 trials. The stimuli were presented in new pseudorandom orders for each participant.

Twenty participants were instructed to indicate right goal side selection by pressing the up-arrow key with the middle finger of their right hand and left goal side selection by pressing the down-arrow key with the index finger of their right hand. The other 20 participants were instructed to indicate right goal side selection by pressing the down-arrow key with the index finger of their right hand and left goal side selection by pressing the upper-arrow key with the middle finger of their right hand. Participants were seated comfortably, aligned centrally to the computer monitor at arm’s length (∼57 cm). In this manner, the goalmouth displacements relative to the center of the computer monitor mirror changes in the participant’s egocentric viewing position of the goalmouth.

### Procedure

At the start of the experiment, participants were presented with written instructions on the computer monitor. From the kicker’s perspective, participants were instructed to decide, as quickly as possible, the best side of the goal (left or right) to place the ball to score a goal. Participants were required to indicate that they had understood the instructions by pressing one of the response keys to start the experimental session. On each trial, each image was presented until the participant made a goal side selection either by pressing the up-arrow key or down-arrow key. Reaction time (RT) was measured from stimulus onset until the participant made a response. For reasons of focus, we do not report the RT data in the present paper. The RT data showed a very similar pattern of results as the binary choice data and beyond methodological interest are superfluous to the main focus of the paper; namely, kicker’s goal side selection. The inter-trial-interval was set at a random duration from 1000 to 3000 ms. On the average, participants took 30 minutes to complete the experiment.

#### Data Analyses

On initial inspection of the data, we removed one participant from the dataset because they consistently chose the left goal side over all the experimental trials. Thereafter, all statistical models were fitted using Bayesian methods as advocated by McElreath (2020). In this regard, all parameter estimates provide for reliability measures in the form of credible intervals (CIs), here determined by the 95% highest probability density interval of each parameter estimate.

To examine the influence of changes in the goalkeeper’s position and goalmouth displacements, we regressed participant’s binary goal side selections (*GS*_01_) on the goalkeeper’s positions (*Keeper*_j_), relative to the center of the goalmouth, and goalmouth location, relative to participant’s body midline (*Kicker*_j_). We entered each participant’s data with their own intercept as well as slopes for the effects of the goalkeeper’s position and kicker’s position. This hierarchical linear model was formulated as follows,

*GS*_01_ ∼ Bernoulli (*p*) [likelihood]

logit (*p*) = *b*_0[subj[i]]_ + *b*_1[subj[i]]_*Keeper*_j_ - *b*_2[subj[i]]_*Kicker*_j_ [linear model]

(*b*_0subj[i],_ *b*_1subj[i],_ *b*_2subj[i]_) ∼ Normal (μ, σ) [*b*_0_, *b*_1_, *b*_2_, priors]

(*b*_0_μ, *b*_1_μ, *b*_2_μ) ∼ Normal (0, 1)  [μ prior]

(*b*_0_σ, *b*_1_σ, *b*_2_σ) ∼ HalfCauchy (0, 1)  [σ prior]

As compared to a comparable intercept only model, successive inclusion of the keeper’s position and kicker’s position improved model prediction; WAIC = 12496, 11157, 10730 respectively. Subsequent inclusion of response assignment or sex (male, female) yielded no further improvement in model fit; WAIC = 10730 respectively.

To examine the joint effect of changes in the goalkeeper’s position and kicker’s position, we regressed participants’ binary goal side choices (*GS*_01_) on the difference between the goalkeeper’s position and kicker’s position (*Keeper*_j_ - *Kicker*_j_) and on the sum of their positions (*Keeper*_j_ + *Kicker*_j_). Again, we entered each participant’s data with their own intercept as well as slope for each effect, by way of the following linear model,

*GS*_01_ ∼ Bernoulli (*p*_i_) [likelihood]

logit (*p*_i_) = *b*_0[subj[i]]_ + *b*_1[subj[i]]_ (*Keeper*_j_ - *Kicker*_j_)+ *b*_2[subj[i]]_ (*Keeper*_j_ + *Kicker*_j_) [linear model]

(*b*_0subj[i],_ *b*_1subj[i],_ *b*_2subj[i]_) ∼ Normal (μ, σ) [*b*_0_, *b*_1_, *b*_2_, priors]

(*b*_0_μ, *b*_1_μ, *b*_2_μ) ∼ Normal (0, 1)  [μ hyper prior]

(*b*_0_σ, *b*_1_σ, *b*_2_σ) ∼ HalfCauchy (0, 1)   [σ hyper prior]

As compared to the intercept only model, inclusion of the difference between the goalkeeper’s position and kicker’s position (*Keeper*_j_ - *Kicker*_j_) and the sum of their positions (*Keeper*_j_ + *Kicker*_j_) successively improved model prediction; WAIC = 12496, 11369, 10730 respectively. Subsequent inclusion of response assignment or sex (male, female) again yielded no further improvement in model fit; WAIC = 10730 respectively.

All data analyses were conducted using R (R Core Team, 2020) and Stan (Carpenter et al., 2017; Stan Development Team, 2020), along with the ‘rethinking’ package (McElreath, 2020). All posterior distributions were based on 2000 warmup steps, and 3000 sampled steps, for each of three independent chains, which showed little autocorrelation. Good convergence obtained between the multiple chains as tested with the *R^* statistic (Gelman et al., 2013), which was found to be less than 1.01 in every case. The resulting Hamilton Monte Carlo samples were therefore highly representative of the underlying posterior distributions. Extensive sensitivity analysis using different priors made no difference to interpretation of the results obtained.

## Results: Study I

### Goalkeeper’s Position and Goalmouth Displacements

**Figure 3.**
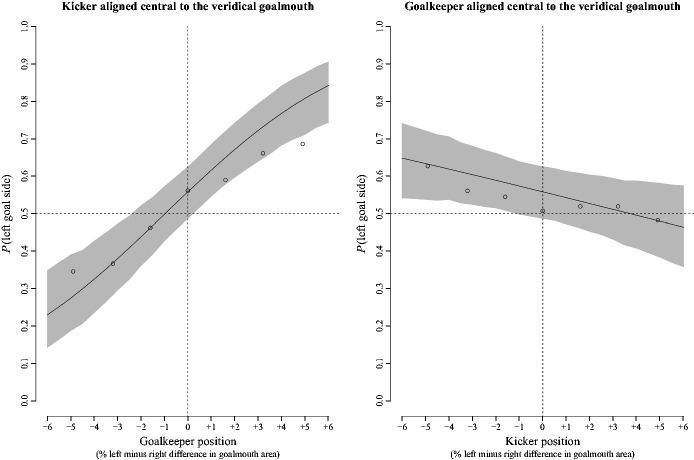
Left Panel: Counterfactual Plot of the Probability (Inverse Logit *P*) of Left Goal Side Selection With Changes in the Goalkeeper’s Position Relative to the Center of the Goal When the Kicker’s Egocentric Viewing Position Was Held at a Constant Position Aligned Central to the Goalmouth. Right Panel: Counterfactual Plot of the Probability of Left Goal Side Selection With Changes in the Kicker’s Egocentric Viewing Position Relative to the Center of the Goalmouth When the Goalkeeper Was Held at a Constant Position Aligned Central to the Goalmouth. *Note.* The unfilled circles show the probability of left goal side selection computed over all participants and marginalized over the kicker’s position (left panel). The unfilled circles show the probability of left side selection computed over all participants and marginalized over the goalkeeper’s position (right panel). In each panel, the solid dark line shows the predicted group level estimate and the shaded area shows the 95% CI about the estimate.

For logistic regression of binary responses on the goalkeeper’s position and kicker’s position group level estimates of the coefficients were: *b*_0_ = 0.23, *b*_1keeper_ = 0.24, *b*_2kicker_ = 0.06, 95% CIs [−0.12, 0.59], [0.13, 0.35], and [−0.01, 0.14], respectively. Figure 3 shows so called counterfactual plots (following McElreath, 2020) of predicted group level estimates of the percentage of left goal side selections, given changes in the goalkeeper’s position (left panel), and given changes in the kickers’ position (right panel).

### Joint Effects of Goalkeepers’ and Kickers’ Positions

**Figure 4.**
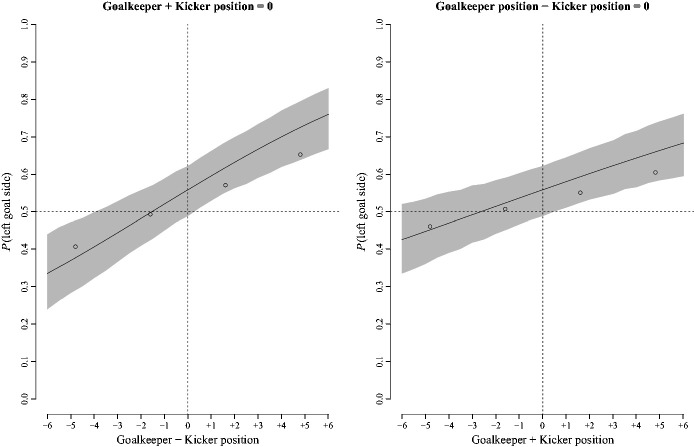
Left Panel: Counterfactual Plot of the Probability of Left Goal Side Selection With Changes in the Difference Between the Goalkeeper’s and Kicker’s Positions When the Joint Sum of their Positions Was Held Constant. Right Panel: Counterfactual Plot of the Probability of Left Goal Side Selection With Changes in the Sum of the Goalkeeper’s and Kicker’s Positions When the Difference in their Positions Was Held Constant. *Note.* The unfilled circles show the probability of left side selection computed over all participants and marginalized over the sum of the goalkeeper’s position and kicker’s position (left panel). The unfilled circles show the probability of left side selection computed over all participants and marginalized over the difference of the goalkeeper’s position and kicker’s position (right panel). In each panel, the solid dark line shows the predicted group level estimate and the shaded area shows the 95% CI about the estimate.

For this logistic regression of binary responses on the relative joint positions of the goalkeeper and kicker, group level estimates of the coefficients were *b*_0_ = 0.23, *b*_1(_*_Keeper_*_−_*_Kicker_*_)_ = 0.15, *b*_2(_*_Keeper_*_+_*_Kicker_*_)_ = 0.09, 95% CIs [−0.11, 0.57], [0.08, 0.23], and [0.03, 0.15], respectively. Figure 4 shows counterfactual plots of predicted group level estimates of the percentage of left goal side selections given changes in the relative difference between the goalkeeper’s position and kicker’s position (left panel), and given changes in the joint sum of the goalkeeper’s and kicker’s position (right panel).

## Discussion: Study I

In Study I, we examined the binary goal side selection of penalty shots in a soccer laboratory experiment, given small changes in the lateral position of the goalkeeper and egocentric viewing position of the kicker (i.e., participant) relative to the veridical center of the goalmouth, manipulating both in a semi-factorial design. Overall, participants tended to choose the left over the right goal side, but this choice depended on the goalkeeper’s lateral position and, to a lesser extent, on the kicker’s egocentric viewing position. Participants’ tendency to choose the left over the right goal side decreased as the goalkeeper shifted leftward and the kicker rightward. Moreover, participants increasingly selected the left goal side as the average position of the goalkeeper and kicker became more rightward: participants’ binary goal side selections depended on the relative positioning of the two soccer players, with the goalkeeper’s position more than twice as important as the kicker’s position in determining the kicker’s goal side selection.

In Study I, the monotonic increase in left goal side selections with small changes in the position of the goalkeeper from left to right of central, conforms to studies of the off-center effect in soccer in which participants have been found to choose the goal side with the greater area to the side of the goalkeeper (Masters et al., 2007; Memmert et al., 2020; Noël et al., 2016; Noël, van der Kamp, & Memmert, 2015; Noël, van der Kamp, Weigelt, et al., 2015; Weigelt & Memmert, 2012; Weigelt et al., 2012). In line with studies of the off-center effect in soccer, in which participants have mostly been young men or women aged in their early twenties, and in which no sex differences in goal-side selection have been previously reported (Memmert et al., 2020; Noël et al., 2016; Noël, van der Kamp, & Memmert, 2015; Noël, van der Kamp, Weigelt, et al., 2015; Weigelt & Memmert, 2012; Weigelt et al., 2012), Study 1 shows no predictive value of including sex (male, female) in our regression model.

In line with studies of the Landmark task (Gökaydin et al., 2017; Märker et al., 2019; McCourt & Olafson, 1997; Toraldo et al., 2004), the estimated PSE, from our logistic regression model, was located just to the left of true center, which cannot simply be attributed to response bias: our analysis shows no predictive value of including our manipulation of response assignment in the regression model. On this basis, Study 1 results suggested that participants tended to overestimate the area to the left, as compared to right, of the goalkeeper’s veridical midline. Consequently, in line with studies of line bisection, small leftward displacements of the goalkeeper from central may actually put the goalkeeper at a disadvantage by increasing the likelihood that kickers will equally, and randomly, choose either goal side to shoot the ball.

Study I findings were in line with results obtained in studies using the Landmark Task (e.g., McCourt & Olafson, 1997), and in line with studies of the off-center effect in soccer (e.g., Masters et al., 2007), but the precise extent to which insights from Study I (a laboratory experiment) generalize to penalty shots in elite soccer matches remains unclear, especially because Study I did not incorporate a team based competitive element and none of Study I participants were experienced soccer players. On this basis, it is pertinent to analyze actual penalty kicks taken in men’s FIFA World Cup matches.

## Method: Study II

### Study II Description

Beyond the work of Masters et al. (2007) who merely reported the proportion of penalty shots to the goal side with the greatest area, detailed archival research has yet to be conducted examining the kicker’s goal side placement of the ball in relation to the goalkeeper’s position and in relation to the kicker’s starting position in elite soccer competitions. On these grounds, in Study II, we set out to examine penalty shots made by professional soccer players under competitive conditions, especially goal side placement of the ball in relation to the position of the goalkeeper (relative to the center of the goal), and initial starting position of the kicker (relative to the center of the goal). Other descriptive aspects of the penalty shots are summarized for general interest.

### Data for Analysis

All Study II analyses were based on video footage of 30 FIFA Men’s World Cup penalty shoot outs (i.e., kicks from the penalty mark taken when one team must be awarded victory and the score is tied after regulation playing time) from 1982 (when the penalty shoot-out was first introduced as a tiebreaker in the FIFA Men’s World Cup) to 2018. All video footage was obtained freely from FIFA online video archives < www.fifa.com >. Video footage of the penalty shots used for the present analysis included: (a) footage showing both the kicker and goalkeeper at their respective starting positions, (b) images displaying the moment at which the ball crossed the goalmouth line, and (c) footage showing whether the penalty shot resulted in a goal or not. Video footage shot from a perspective that did not allow for measurement of the goalkeeper’s starting position and kicker’s initial position was excluded from analysis. On this basis, one hundred penalty kicks were selected for analysis.

For measurement of the starting position of the goalkeeper’s position and kicker’s position, individual video frames were extracted at a rate of 60 per second using the free and open source VL media player (VideoLAN organization, France). Each image was scaled to a pixel resolution of 1250×927 on a Hewlett Packard 450 G5 Notebook PC (Hewlett Packard Enterprise, USA), with a 39.6 cm (15.6 in) diagonal screen with an active area of 344.2×193.5 mm. The pixel resolution of the video monitor was 1366×768, and so the viewable size of each image on the computer screen was 315×233.6 mm.

All measurements were done manually by placing gridlines over each image using the ruler facilities provided by Microsoft PowerPoint (Microsoft Corporation, 2016). Measurements of interest were the goalkeeper’s initial displacement relative to the center of the goal (veridical center of the goalkeeper to the veridical center of the goalmouth), kicker’s starting position relative to the center of the goal (veridical center of the kicker relative to the veridical center of the goalmouth), and for scaling purpose the goalmouth width represented in each image. All measurements were taken in millimeters, converted to centimeters and subsequently scaled to real size goal dimensions. Other aspects of interest were the foot used by the kicker to take the penalty shot, whether the goalkeeper dived to the left or right, and whether the penalty shot resulted in a goal or not.

#### Data Analyses

Overall, 76% of the penalty shots resulted in a goal. The goalkeeper’s starting position was to the right of the center of the goalmouth on 62% of the penalty shots and the goalkeeper dived to the left on 54% of the penalty shots. The kicker’s starting position was to the left of the ball 77% of the time. The kicker directed the ball to the goal side with the greatest area to the side of the keeper on 51% of the penalty shots. On 76% of the penalty shots the kicker took the penalty shot with their right foot and only one occasion occurred in which the kicker took the penalty shot using the foot corresponding to their starting position – in this case the kicker started their run up to the ball standing to the left of the ball and used their left foot to kick the ball. On 55% of the penalty shots the kicker kicked the ball to the left side of the goalmouth, but the goalkeeper dived to the opposing side of the kicker’s ball placement 47% of the time (i.e., the kicker kicked the ball to the right or left of the goalkeeper and the goalkeeper dived to left or right, respectively).

To examine relations between kickers’ goal side selection, position of the goalkeeper (relative to the center of the goal), and starting position of the kicker (relative to the center of the goal), we regressed each left or right goal side placed penalty shot on the goalkeeper’s position and kicker’s position. In this case, each kicker made only one penalty shot and so a hierarchical modelling approach is ruled out, but otherwise this analysis followed the same procedures as described in Study I,

*GS*_01_ ∼ Bernoulli (*p*) [likelihood]

logit (*p*) = *b*_0_ + *b*_1_*Keeper*_j_ + *b*_2_*Kicker*_j_ [linear model]

(*b*_0,_ *b*_1,_ *b*_2_) ∼ Normal (0, 1)   [intercept and slope priors]

As compared to an intercept only model, successive inclusion of the goalkeeper’s position and kicker’s position failed to improve model fit; WAIC = 140, 141, 143 respectively.

Following the procedures detailed in Study I, joint effects of the goalkeeper’s position and kicker’s position on goal side selection were examined by regressing each goal side selection on the difference between the goalkeeper’s position and kicker’s position and on the sum of their positions.

*GS*_01_ ∼ Bernoulli (*p*) [likelihood]

logit (*p*) = *b*_0_ + *b*_1_ (*Keeper*_j_ - *Kicker*_j_)+ *b*_2_ (*Keeper*_j_ + *Kicker*_j_) [linear model]

(*b*_0,_ *b*_1,_ *b*_2_) ∼ Normal (0, 1)   [intercept and slope priors]

As compared to an intercept only model, successive inclusion of the difference between the goalkeeper’s position and kicker’s position (*Keeper*_j_−*Kicker*_j_) and the sum of their positions (*Keeper*_j_ + *Kicker*_j_) failed to improve model fit; WAIC = 140, 141, 143 respectively.

Exactly, the same procedures were used to fit the models in Study II as described in Study I. All HMC chains showed good convergence, and extensive sensitivity analysis using different priors made no difference to interpretation of the final results obtained.

## Results: Study II

### Goalkeepers’ Position and Goalmouth Displacements

**Figure 5.**
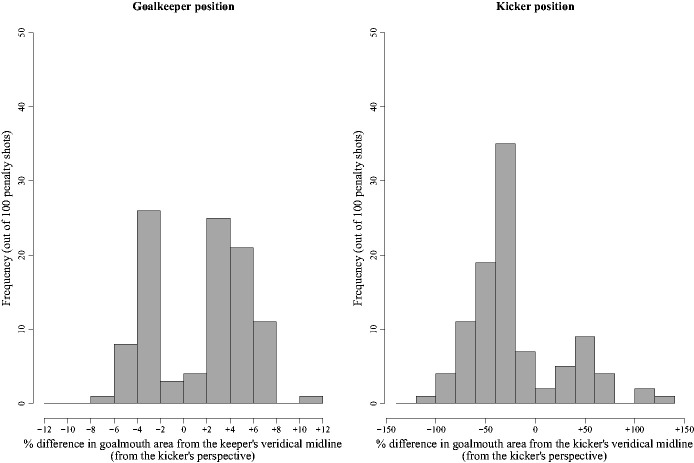
Frequency Histograms Showing the Goalkeeper’s Position (left panel) and Kicker’s Position (Right panel) for Each of the 100 Penalty Shots Analyzed. *Note.* On the rare occasion in which the kicker’s position is greater than ±100%, the kicker’s initial standing position, before making their run up to the ball, was beyond the extent of the vertical left or right goalpost.

Figure 5 shows histograms of the frequency (out of 100) of the goalkeeper’s position and kicker’s position. On the mean average, the goalkeeper tended to stand to the right of the center of the goalmouth (*M* ± *SD* = 1.49 ± 4.1%), and the kicker’s starting position was most often to the left of the ball, (*M* ± *SD* = −21.02 ± 47.1%).

**Figure 6.**
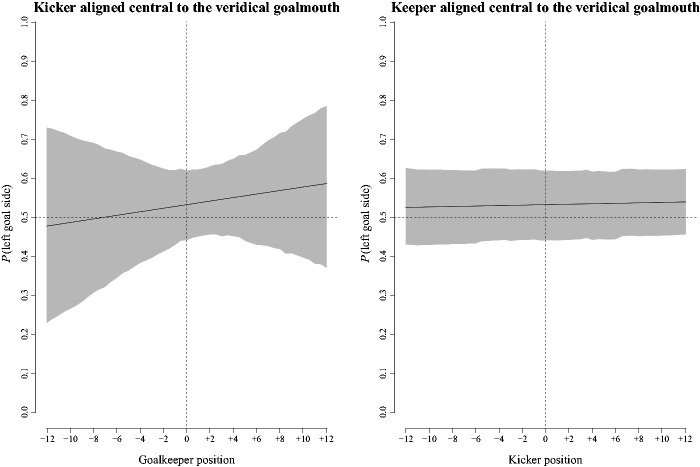
Left Panel: Counterfactual Plot of the Probability of Left Goal Side Ball Placement With Changes in the Goalkeeper’s Position Relative to the Center of the Goal When the Kicker’s Egocentric Viewing Position Was Held at a Constant Position Aligned Central to the Goalmouth. Right Panel: Counterfactual Plot of the Probability of Left Goal Side Ball Placement With Changes in the Kicker’s Egocentric Viewing Position Relative to the Center of the Goalmouth When the Goalkeeper Was Held at a Constant Position Aligned Central to the Goalmouth. *Note*. In each panel, the solid dark line shows the predicted group level estimate, and the shaded area shows the 95% CI about the estimate.

Logistic regression analyses in Study II, showed a very small and considerably variable effect of the goalkeeper’s position on the kicker’s goal side ball placement. No relation was found between the kicker’s initial starting position and their subsequent goal side placement of the ball. Logistic regression estimates of the coefficients were *b*_0_ = 0.13, *b*_1Keeper_ = 0.02, *b*_2Kicker_ = 0.00, 95% CIs [−0.32, 0.56], [−0.08, 0.11], and [−0.01, 0.01], respectively. Figure 6 shows counterfactual plots of predicted estimates of the percentage of left goal side shots, given changes in the goalkeeper’s position, and given changes in the kicker’s position.

### Joint Effects of Goalkeeper and Kickers’ Positions

The joint analysis of goalkeepers’ and kickers’ positions showed a very small but increasing tendency for professional male footballers to shoot the ball to the left goal side as the goalkeeper stood further to the right of the veridical center of the goalmouth and the kicker initially stood at a position increasingly left of the ball. Moreover, the kickers tended to kick the ball more often to the left of the veridical center of the goalmouth as the joint average position of both players became increasingly rightward. In line with Study 1, the indication is that the kickers’ goal side selection is related to the relative positioning of the 2 soccer players. However, in analysis of these world cup penalty shots, the joint effects of the goalkeeper’s position and kicker’s position are very small and unreliable.

**Figure 7.**
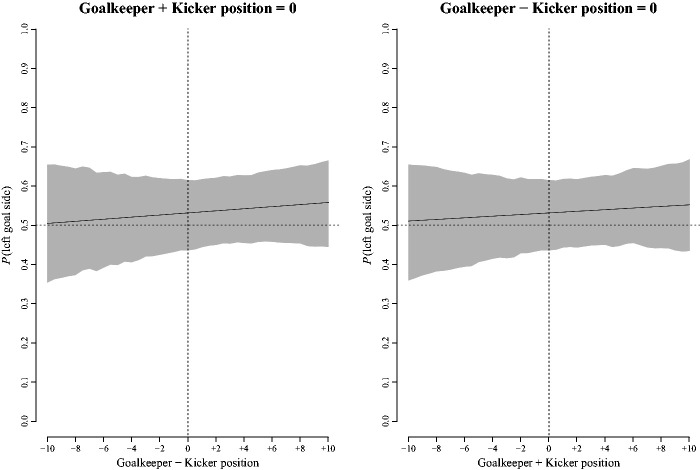
Left Panel: Counterfactual Plot of the Probability of Left Goal Side Ball Placement With Changes in the Difference Between the Goalkeeper’s and Kicker’s Positions When the Joint Sum of Their Positions Was Held Constant. Right Panel: Counterfactual Plot of the Probability of Left Goal Side Ball Placement With Changes in the Sum of the Goalkeeper’s and Kicker’s Positions When the Difference in Their Positions Was Held Constant. *Note.* In each panel, the solid dark line shows the predicted group level estimate and the shaded area shows the 95% CI about the estimate.

The logistic regression of binary responses on the relative joint positions of the goalkeeper and kicker yielded the following coefficient estimates, *b_0_* = 0.12, *b*_1(_*_Keeper_*_−_*_Kicker_*_)_ = 0.01, *b*_2(_*_Keeper_*_+_*_Kicker_*_)_ = 0.01, 95% CIs [−0.32, 0.57], [−0.04, 0.06], and [−0.04, 0.06], respectively. Figure 7 shows counterfactual plots of the estimated percentage of left goal side shots, given changes in the relative difference between the goalkeeper’s position and kicker’s position, and given changes in the sum of the goalkeeper’s and kicker’s positions.

## Discussion: Study II

In Study II, we examined actual kickers’ goal side selection of penalty shoot-outs in men’s FIFA World Cup soccer matches. In line with Study I these data showed a small tendency for kickers to shoot the ball to the left goal side more often than the right goal side. This leftward tendency conforms to earlier analyses of elite football competitions in which kickers were also found to direct their penalty shots more often to the left than right goal side (Bar-Eli & Azar, 2009; Price & Wolfers, 2014; Roskes et al., 2011). However, as mirrored in the wide credible intervals obtained in the present analysis, and as reported by others (e.g., Avugos et al., 2020), this tendency for kickers to shoot the ball to the left was highly variable and may have sometimes crossed over to become rightward.

Regarding the off-center effect in soccer, our data showed that actual kickers tended to select the left, as compared to right, goal side increasingly more often as the goalkeeper was increasingly positioned to the right of the true center veridical center of the goalmouth. This finding fits with Study I, but in contrast to Study I, in actual games this off-center effect was very small and highly variable, with no main effect obtained for changes in the kicker’s position. Moreover, joint effects of changes in both the goalkeeper’s and kicker’s position, on the kicker’s goal side selection where similarly found to be very small and highly variable. In all, Study II findings suggest that kickers’ goal side selection of penalty shots in world cup matches were barely influenced by small displacements of the goalkeeper from central, were not influenced by the kicker’s starting position, and were only vaguely influenced by the joint position of both the goalkeeper and kicker. All of these effects were found to be small and highly variable, Study I.

## General Discussion

In Study I, our participants (naïve to soccer) preferred the left, as compared to right, goal side for scoring a goal with a penalty kick, but this left side preference was modulated when both the positions of the goalkeeper and kicker were systematically displaced from central in semi-factorial combination. Overall, Study I findings conform to those obtained in studies of line bisection, especially the Landmark Task, in which neurological healthy participants (a) typically show a small tendency to bisect lines to the left of center (Jewell & McCourt, 2000; Milner et al., 1992), (b) increasingly judge the left segment of bisected lines, as compared to the right, as longer, as the transection mark is moved from left to right of central (Gökaydin et al., 2017; Märker et al., 2019; McCourt & Olafson, 1997; Toraldo et al., 2004) and, (c) with increasing displacement of lines from participant’s body midline, increasingly bisect lines to the side corresponding to their egocentric viewing position (Bultitude & Davies, 2006; McCourt & Jewell, 1999; Mennemeier et al., 2001; Reuter-Lorenz et al., 1990; Rinaldi et al., 2018; Zago et al., 2017).

Study II built on Study I by examining the influence of the goalkeeper’s and kicker’s positions on the kicker’s goal side selection in actual world cup shoot-outs. In line with earlier studies (Bar-Eli & Azar, 2009; Price & Wolfers, 2014; Roskes et al., 2011), Study II revealed a tendency for kickers to start their run-up to the ball from their left and showed a slight, but highly variable, tendency for kickers to shoot the ball to the left goal side. In terms of the biomechanics of kicking, most soccer players find it easier to use the inside of their left or right dominant foot to kick the ball to their right or left, respectively (Chiappori et al., 2002; Palacios-Huerta, 2003). But, most elite soccer players can also use their non-dominant, left or right, foot to kick the ball, and beyond the biomechanics of kicking perceptual and cognitive factors may also play a role in kickers’ goal side selections.

Study I, like studies of line bisection, suggests that participants tended to overestimate the goalmouth area to the left, as compared to right, of the goalkeeper’s midline. This contrasts with studies conducted by Noël, van der Kamp, and Memmert (2015) and Noël, van der Kamp, Weigelt, et al. (2015) who found a tendency for participants to position the goalkeeper just to the right of central when instructed to position the goalkeeper centrally. Although Noël, van der Kamp, & Memmert (2015) and Noël, van der Kamp, Weigelt, et al. (2015) found a slight tendency for kickers to aim the ball to the left goal side, others (Memmert et al., 2020; Weigelt & Memmert, 2012; Weigelt et al., 2012) have reported a small tendency for participants to select the right goal side when taking penalty shots. Overall, this goal side selection variability in studies of the off-center effect in soccer mirrors findings in line bisection research in which both the magnitude and direction of line bisection errors have been shown to be highly susceptible to individual differences and manipulation of perceptual aspects of the lines, such as viewing distance and spatial locations of the lines (Jewell & McCourt, 2000; McCourt & Olafson, 1997).

### Implications

On the basis that kickers tend to select the goal side with the greatest area, Masters et al. (2007) and others (Memmert et al., 2020; Noël et al., 2016; Noël, van der Kamp, & Memmert, 2015; Noël, van der Kamp, Weigelt, et al., 2015; Weigelt & Memmert, 2012; Weigelt et al., 2012) suggested that goalkeepers may gain an advantage in penalty kick situations by standing marginally off-center to influence kickers’ goal side selections. Our findings, however, in connection with line bisection research, suggest that a goalkeeper positioned marginally to the left or right of the center of the goalmouth may actually lead to kickers’ choosing more equally and randomly to shoot the ball to the left or right goal side, reducing the predictability of the kicker’s goal side selections. In Study II, based on actual soccer world cup competition as opposed to a laboratory experiment, the off-center effect was barely existent and highly variable.

Further research analyzing the behavior of goalkeepers and kickers in elite soccer competitions is required to corroborate the findings of the present study. But, on the basis of the present study, soccer related experimental tasks, conducted under controlled laboratory conditions, do not necessarily reflect the behavior of skilled players in elite matches. In elite soccer games the penalty kick pits the will of two highly skilled players against each other, while both try to outwit each other. While neuropsychological studies of line bisection have potential to inform us about the soccer players’ behavior, neurological studies of line bisection typically lack any competitive element.

### Limitations and Future Directions

Future studies of goal side selection in soccer may benefit from physically varying the kicker’s starting position, relative to veridical center of the goalmouth, and distance of the kicker from the goal-line. In Study I, displacement of the goalmouth relative to the center of the computer screen, necessarily reduced the distance of the left or right goalpost from the edge of the computer screen. This alone may have induced an apparent effect of changes in the kicker’s position, that was otherwise not found in Study II. More detailed analyses of kickers’ behavior, in the laboratory and in elite soccer competitions, may provide useful clues a goalkeeper might use to save penalty kicks, such as the kicker’s starting distance from the ball, speed with which a kicker takes the penalty shot, and individual player’s history of penalty shots in world cup matches.

## Conclusions

Under laboratory controlled experimental conditions, the off-center effect in soccer yielded results comparable to those obtained in studies of line bisection, especially the Landmark Task. However, parallel data from world cup soccer matches regarding actual player behavior in penalty goal kicks differed from these results, suggesting that the experimental soccer related task did not generalize well to the behavior of highly skilled goalkeepers and kickers in actual matches. Great care should be exercised when making bench side recommendations about the behavior of skilled soccer players on the basis of controlled experimental tasks.

## Notes

**Author Biographies:** **Mauro R. Pereira** is currently pursuing a Master of Science degree in Psychology at Lund University and is interested in psychophysics and soccer coaching. **Geoffrey R. Patching** is a professor of Cognitive Psychology at Lund University interested in psychophysics, human information processing, and Bayesian statistics.
